# Targeted inhibition of CX3CL1 limits podocytes ferroptosis to ameliorate cisplatin-induced acute kidney injury

**DOI:** 10.1186/s10020-023-00733-3

**Published:** 2023-10-24

**Authors:** Qiming Gong, Tengfang Lai, Liudan Liang, Yan Jiang, Fahui Liu

**Affiliations:** 1 Department of Nephrology, Affiliated Hospital of Youjiang Medical University for Nationalities, No.18 Zhongshan Road, Baise, 533000 Guangxi China; 2 Department of Cardiology, Affiliated Hospital of Youjiang Medical University for Nationalities, Baise, 533000 Guangxi China; 3 Department of Infection, Affiliated Hospital of Youjiang Medical University for Nationalities, Baise, 533000 Guangxi China; 4 The Key Laboratory for High Incidence Prevention and Treatment in Guangxi Guixi Area, Youjiang Medical University for Nationalities, Baise, 533000 Guangxi China; 5 Xiamen Cell Therapy Research Center, The First Affiliated Hospital of Xiamen University, School of Medicine, Xiamen University, Xiamen, 361003 China

**Keywords:** CX3CL1, Cisplatin, Acute kidney injury, Podocyte, Ferroptosis

## Abstract

**Background:**

It is widely acknowledged that cisplatin-induced nephrotoxicity hinders its efficacy during clinical therapy. Effective pharmaceutical interventions for cisplatin-induced acute kidney injury (Cis-AKI) are currently lacking. Prior studies have implicated the chemokine CX3CL1 in the development of lipopolysaccharide-induced AKI; however, its specific role in Cis-AKI remains uncertain. This research aimed to comprehensively characterize the therapeutic impact and mechanism of CX3CL1 inhibition on Cis-AKI.

**Methods:**

This study employed an in vivo Cis-AKI mouse model and in vitro cisplatin-treated podocytes. Kidney pathological changes were assessed using hematoxylin–eosin (HE) and Periodic-Schiff (PAS) staining. Transcriptome changes in mouse kidney tissue post-cisplatin treatment were analyzed through RNA sequencing (RNA-seq) datasets. Evaluation parameters included the expression of inflammatory markers, intracellular free iron levels, ferroptosis-related proteins—solute carrier family 7 member 11 (SLC7A11/XCT) and glutathione peroxidase 4 (GPX4)—as well as lipid peroxidation markers and mitochondrial function proteins. Mitochondrial morphological changes were visualized through transmission electron microscopy. The impact of CX3CL1 on the glucose-regulated protein 78/eukaryotic translation initiation factor 2A/CCAAT enhancer binding protein-homologous protein (GRP78/eIF2α/CHOP) and hypoxia-inducible factor 1-alpha/heme oxygenase-1 (HIF1A/HO-1) pathways in Cis-AKI was assessed via Western Blot and Immunofluorescence experiments, both in vivo and in vitro.

**Results:**

Kidney CX3CL1 levels were elevated following cisplatin injection in wild-type (WT) mice. Cisplatin-treated CX3CL1-Knockout mice exhibited reduced renal histological changes, lowered blood creatinine (Cre) and blood urea nitrogen (BUN) levels, and decreased expression of inflammatory mediators compared to cisplatin-treated WT mice. RNA-seq analysis revealed the modulation of markers associated with oxidative stress and lipid metabolism related to ferroptosis in the kidneys of mice with Cis-AKI. Both the in vivo Cis-AKI mouse model and in vitro cisplatin-treated podocytes demonstrated that CX3CL1 inhibition could mitigate ferroptosis. This effect was characterized by alleviated intracellular iron overload, malondialdehyde (MDA) content, and reactive oxygen species (ROS) production, alongside increased glutathione/glutathione disulfide ratio, superoxide dismutase (SOD), XCT, and GPX4 activity. CX3CL1 inhibition also ameliorated mitochondrial dysfunction and upregulated expression of mitochondrial biogenesis proteins-uncoupling protein (UCP), mitofusin 2 (Mfn2), and peroxisome proliferators-activated receptor γ coactivator l-alpha (PGC1α)-both in vivo and in vitro. Furthermore, CX3CL1 inhibition attenuated cisplatin-induced endoplasmic reticulum (ER) stress in podocytes. Notably, CX3CL1 inhibition reduced cisplatin-induced expression of HIF-1α and HO-1 in vivo and in vitro.

**Conclusion:**

Our findings suggest that CX3CL1 inhibition exerts therapeutic effects against Cis-AKI by suppressing podocyte ferroptosis.

## Introduction

Acute kidney injury (AKI) is characterized by a sudden decline in renal function, accompanied by structural and functional kidney impairment, leading to substantial morbidity and mortality (Ronco et al. 2019). Kidney ischemia, medication toxicity, and exposure to heavy metals are widely acknowledged as the primary causes of AKI development (Kwiatkowska et al. 2021). Cisplatin (Cis), an effective drug for various solid tumors, typically accumulates in the kidneys, resulting in nephrotoxicity that compromises both glomerular and tubular functions (Pabla and Dong 2008). However, viable pharmacological options for managing cisplatin-induced AKI (Cis-AKI) are currently lacking and ineffective.

Although the exact pathophysiology of Cis-AKI remains elusive, specific mechanisms, such as oxidative stress and lipid peroxidation, have been proposed (Sánchez-González et al. 2011; Fang et al. 2021). It is now understood that once cisplatin infiltrates cells, it hampers antioxidant enzymes like glutathione peroxidase, superoxide dismutase (SOD), and glutathione reductase, culminating in elevated reactive oxygen species (ROS) levels that trigger oxidative stress and lipid peroxidation (Jesse et al. 2014). This excess lipid peroxidation precipitates ferroptosis, an iron-dependent form of regulated cell death (Lei et al. 2019). Recent studies revealed a positive correlation between serum iron levels and mortality risk in AKI patients (Shu et al. 2020). Besides, Cisplatin treatment induces serum iron accumulation and kidney cell peroxidation (Ikeda et al. 2021), establishing a close link between Cis-AKI and ferroptosis. Hence, inhibiting ferroptosis represents a promising treatment approach against Cis-AKI.

Chemokines often play a pivotal role in chronic and acute inflammations (Homey et al. 2002). Within the CX3C chemokine family, only one member has been identified so far-CX3CL1, also known as fractalkine or FKN. CX3CL1 serves diverse functions and is expressed in multiple tissues. Studies have demonstrated CX3CL1's role in enhancing lymphocyte and mesenchymal cell migration and chemotaxis. Furthermore, its deficiency has been associated with reduced extracellular matrix deposition in a murine diabetes model induced by streptozotocin (Song et al. 2013). Prior research from our group has implicated CX3CL1 in sepsis-induced AKI and clear cell renal cell carcinoma (ccRCC) (Gong et al. 2021, 2022; Liu et al. 2022). However, the exact relationship between CX3CL1 and ferroptosis in Cis-AKI remains to be established.

Herein, we hypothesized that CX3CL1 deficiency could mitigate ferroptosis, reducing cisplatin-triggered podocyte injury and kidney inflammation. Accordingly, we developed both a Cis-AKI mouse model and a podocyte model. We evaluated key ferroptosis factors-glutathione peroxidase 4 (GPX4) and solute carrier family 7 member 11 (SLC7A11/XCT)-alongside inflammatory markers-interleukin 6 (IL-6) and tumor necrosis factor-α (TNF-α). Additionally, we assessed the expression of mitochondrial biogenesis proteins, glucose-regulated protein 78/eukaryotic translation initiation factor 2A/CCAAT enhancer binding protein-homologous protein (GRP78/eIF2α/CHOP), and hypoxia-inducible factor 1-alpha/heme oxygenase-1 (HIF1A/HO-1) pathways. Our findings indicated that CX3CL1 deficiency could dampen inflammation in Cis-AKI mice. Mechanistically, CX3CL1 deficiency prevented mitochondrial dysfunction, mitigated endoplasmic reticulum (ER) stress, inhibited the HIF1A/HO-1 pathway, and protected against Cis-AKI by suppressing ferroptosis in podocytes. Consequently, targeting CX3CL1 could hold potential as a therapeutic strategy for Cis-AKI.

## Materials and methods

### Development and handling of the animal model

All procedures involving animals were conducted following the Animal Research: Reporting of In Vivo Experiments (ARRIVE) guidelines and were subjected to approval from the animal experimentation ethics of Youjiang Medical University for Nationalities (ethical batch number: 2021103001). The C57BL/6 mice (NO. SYXK 2015-0008) were provided by Shanghai Genechem Animal Co., Ltd. These mice were housed at the SPF facility of Youjiang Medical University for Nationalities (NO. SYXK 2017‐0004) under controlled conditions, including a 12-h light/dark cycle, 50% humidity, and temperatures between 20 and 24 °C. The mice had unrestricted access to sterilized water and food. A CX3CL1-Knockout (CX3CL1-KO) mouse model was established, as indicated in a previous study (Gong et al. 2021). Each mouse was randomly assigned to one of three groups (*n* = 5): control group, cisplatin group, and cisplatin + CX3CL1-KO group. The animals received a single intraperitoneal dose of cisplatin (20 mg/kg) (Zhou et al. 2022). 48 h after receiving cisplatin, mice were humanely killed by cervical dislocation, and samples of blood and renal tissues were obtained.

### Cell culture and treatment

The AB8/13 human immortalized podocyte cell line was generously provided by Dr. Moin A. Saleem from Bristol, U.K. Podocytes were cultured following previously established protocols (Gong et al. 2022). Lentiviral vector Ubi-MCS-CBh-gcGFP-IRES-Puro-CX3CL1 was employed to induce CX3CL1 knockdown (CX3CL1-KD) in podocytes, following the manufacturer’s instructions from Shanghai Genechem Co., Ltd. Three distinct podocyte groups were established: the control group, the cisplatin-treated group (20 μM/ml, 24 h), and the CX3CL1-KD + cisplatin group.

### Bioinformatic analysis

Published RNA-seq data in Total Productive Maintenance (TPM) format were downloaded from the Gene Expression Omnibus (GEO) database (accession number: GSE106993). TPMs were converted to log2 (TPM + 1) for further analysis. Differentially expressed genes (DEGs) between cisplatin-treated and wild-type (WT)-group mice were examined with the limma package. Genes with LogFC > 1.5 and adjusted *p*-value < 0.05 were statistically significant. Functional enrichment analysis was conducted with Gene Ontology (GO) and the Kyoto Encyclopedia of Genes and Genomes (KEGG) for all differential genes. The functional differences between the two treatment groups were further investigated by conducting additional Gene Set Enrichment Analysis (GSEA). The MSigDBR package was used to acquire the hallmark gene set (c2.all.v7.5.1.symbols.gmt).

### Renal function assessment

The serum levels of blood urea nitrogen (BUN) and serum creatinine (Scr) in mice were measured by urease and sarcosine oxidase assays, respectively, according to the manufacturer (Jiancheng Bioengineering Institute, Nanjing, China; kit numbers C013-2-1 and C011-2-1, respectively).

### MDA, SOD, and iron content and GSH/GSSG ratio analysis

The contents of malondialdehyde (MDA), superoxide dismutase (SOD), and iron and the glutathione/oxidized glutathione (GSH/GSSG) ratio in kidney tissues and podocytes were detected using MDA (thiobarbituric acid method) and SOD assay kits (hydroxylamine method; Jiancheng Bioengineering Institute, Nanjing, China), an Iron content assay Kit (ferrozine microplate method; Leagene Biotechnology, Beijing, China), and a GSH/GSSG assay kit (fluorometric—green; ab205811, Abcam), respectively, following the corresponding kit instructions.

### Enzyme-linked immunosorbent assay (ELISA)

The serum levels of IL-6 and TNF-α were quantified using ELISA kits (Cusabio Biotech Co., Ltd) according to the manufacturer’s instructions.

### Hematoxylin and eosin (HE) and periodic acid-Schiff (PAS) staining

The kidney tissues were fixed in 4% formaldehyde and embedded in paraffin. The tissues were then sliced into 4 μm thickness sections for HE and PAS staining. Light microscopy (BX53, Olympus, Tokyo, Japan) was used to study renal tissue pathology. The pathological changes in the kidney tissues were evaluated by a board-certified pathologist. To ensure impartiality, the pathologist was blinded to the treatment conditions during the evaluation, eliminating potential biases. A scoring system (ranging from 0 to 4 points), based on the extent of injury (< 25% injury, 25–50% injury, 50–75% injury, and > 75% injury), was employed to gauge the severity of glomerular sclerosis through PAS staining (Liu et al. 2022).

### Immunohistochemistry (IHC)

Renal tissue section (4 μm) were fixed in 4% formaldehyde. After deparaffinization and rehydration, IHC was performed on the sections using anti-TNF-α, anti-F4-80 (mouse EGF-like module-containing mucin-like hormone receptor-like 1), anti-4-HNE (4-Hydroxynonenal), anti-3-NT (3-nitrotyrosine), or anti-GPX4 primary antibody (all from Affinity Biosciences, OH, USA; 1:200). The primary antibodies were incubated at 4 °C overnight, followed by subsequent incubation with horseradish peroxidase-conjugated secondary antibody (Dako, Glostrup, Denmark) at a 1:200 dilution at room temperature (RT) for 1 h. The sections were washed with PBS following each process. In the absence of the primary antibody or upon the addition of excessive antigen, the staining condition was assessed to ensure specificity. The fluorescence image was captured by a fluorescence microscope (BX53, Olympus, × 400). Staining intensities were quantified with ImageJ software.

### Reactive oxygen species (ROS) analysis

Renal tissue section (4 μm) were first incubated with 20 μM dihydroethidium (DHE-DA; Meilunbio, Dalian, China) for half an hour at RT. The ROS level of podocytes was determined using a 2,7-dichlorodihydrofluorescein diacetate (DCFH-DA) probe (Beyotime, Shanghai, China). To study its effects, the podocytes were first treated with cisplatin; then, they were subjected to incubation at RT for half an hour with 2 μL/well of DCFH-DA. The ROS levels in renal tissues and podocytes were observed using fluorescence microscopy (Olympus, Tokyo, Japan).

### Transmission electron microscopy (TEM)

Fresh renal tissues (1 mm^3^) were placed in a TEM fixative (P1126, Servicebio, Beijing, China) for 2 h at 4 °C. After tissues were sliced into the ultrathin section (60–80 nm), the specimens were stained with uranyl acetate and lead citrate before being examined by TEM (HT7700, HITACHI, Tokyo, Japan).

### Immunofluorescence (IF) staining

Renal cryosections (3 μm) and podocyte sections were fixed using 4% paraformaldehyde (PFA), followed by blocking using 10% fetal bovine serum (FBS). The sections were then immunostained with anti-CX3CL1, anti-nephrin, anti-TNF-α, anti-eIF2α, anti-p-eIF2α, anti-CHOP, anti-HO-1, anti-HIF1A, anti-GPX4 or anti-XCT primary antibody overnight at 4 °C. Next, the sections were subjected to incubation with goat anti-rabbit secondary antibody for an hour. The stained slice images were then captured under a fluorescence microscope (Olympus, Tokyo, Japan). Image pro-plus 6.0 software was used to analyze the integrated optical density (IOD) of each fluorescent image, and finally, the average IOD was calculated.

### Western blotting

Renal tissues and podocytes were first lysed utilizing RIPA solution on ice. The total proteins isolated were then examined using the specific anti-CX3CL1 (1:2000, ab25088, Abcam), anti-nephrin (1:1000, AF7951, Affinity Biosciences), anti-podocin (1:1000, DF8593, Affinity Biosciences), anti-WT1 (Wilms tumor protein; 1:800, DF6331, Affinity Biosciences), anti-TNF-α (1:1000, AF7014, Affinity Biosciences), anti-IL-6 (1:1000, DF6087, Affinity Biosciences), anti-F4/80 (1:750, DF2789, Affinity Biosciences), anti-UCP2 (uncoupling protein 2; 1:750, DF8626, Affinity Biosciences), anti-Mfn2 (Mitofusin 2; 1:800, DF8106, Affinity Biosciences), anti-PGC1α (peroxisome proliferators-activated receptor γ coactivator lalpha; 1:1000, Af5395, Affinity Biosciences), anti-GPX4 (1:1000, DF6701, Affinity Biosciences), anti-XCT (1:1000, DF12509, Affinity Biosciences), anti-GRP78 (1:1000, BF8024, Affinity Biosciences), anti-eIF2α (1:1000, AF6216, Affinity Biosciences), anti-p-eIF2α (1:1000, AF3216, Affinity Biosciences), anti-CHOP (1:1000, AF6277, Affinity Biosciences), anti-HO-1 (1:750, AF5393, Affinity Biosciences), anti-HIF1A (1:1500, ab179483, Abcam) and anti-β-actin (1:5000, AF7018, Affinity Biosciences) primary antibody followed by IRDye 800RD goat anti-rabbit (SA535571, Invitrogen, USA) secondary antibody. Stained proteins were then detected using a Licor Odyssey scanner (Licor Bioscience, Lincoln, USA).

### Mitochondrial membrane potential (MMP) measurement

The JC-1 fluorescence staining assay was conducted to determine the MMP. Briefly, podocytes were harvested and incubated with JC-1 (Sigma-Aldrich, MO, USA) for 20 min at RT. Podocyte fluorescence intensity was then measured using fluorescence microscopy (Olympus, Tokyo, Japan). The presence of red fluorescence within podocytes indicated the aggregation dependent on mitochondrial potential, reflecting the mitochondrial membrane potential (ΔΨm). A transition from red to green fluorescence indicated depolarization in the mitochondria.

### Statistical analysis

Data were presented as mean ± standard deviation (SD). GraphPad Prism 8 was utilized for statistical analyses. The statistical significance of the difference (variation) between groups was assessed using the Student’s two-tailed unpaired *t*-test and one-way analysis of variance (ANOVA). Tukey’s test was performed for multiple comparisons. *p* values < 0.05 indicated statistically significant differences between groups.

## Results

### CX3CL1 knockout inhibited podocyte injury caused by cisplatin in mouse kidney

**Fig. 1:**
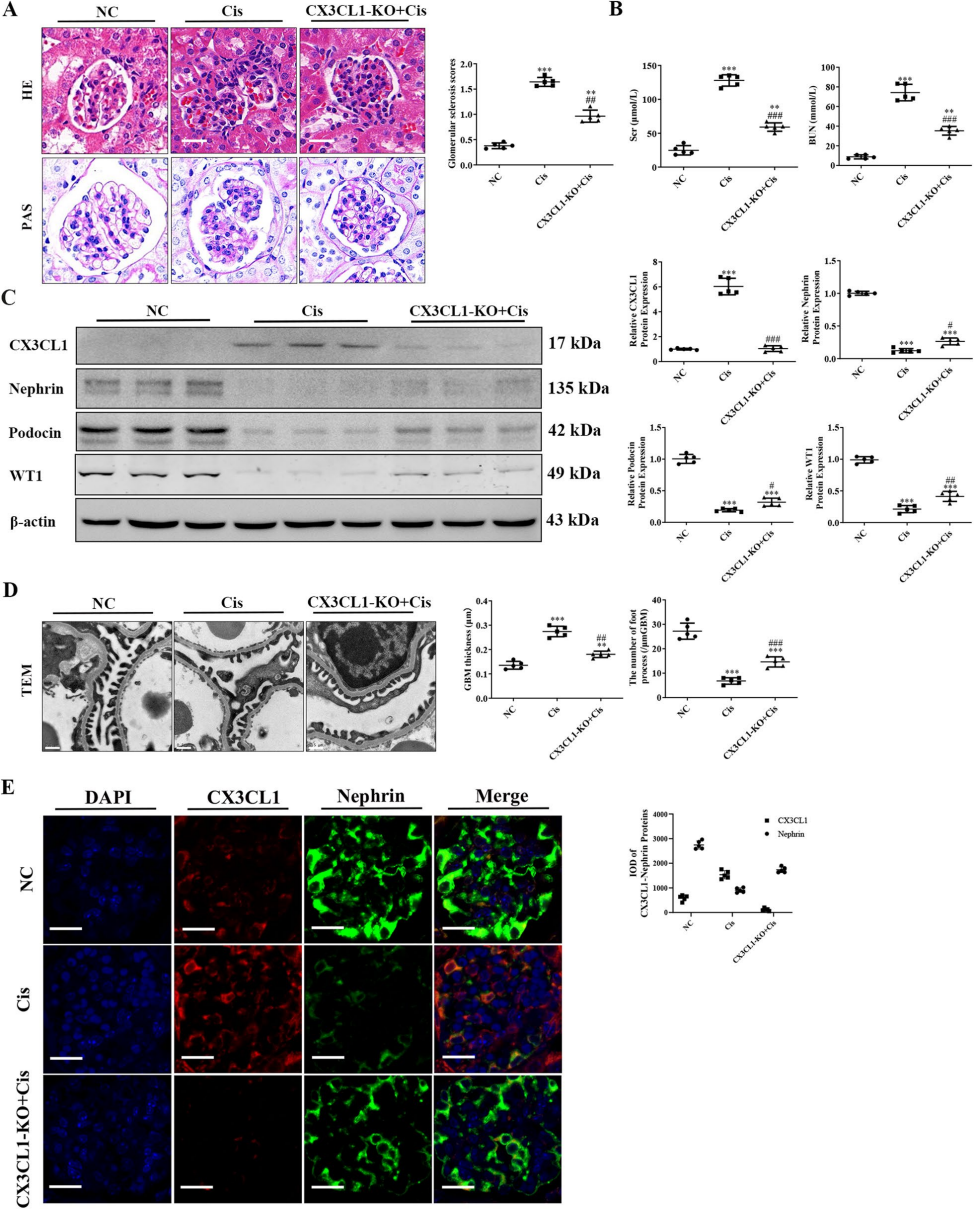
CX3CL1 deficiency alleviated kidney function damage in cisplatin-induced model mice. WT mice and CX3CL1-KO mice received cisplatin injections at 48 h. **A** Examination of the kidney’s histology using representative images of H&E and PAS staining. **B** The serum levels of BUN and Scr were evaluated in all mice groups. **C** Western blotting was conducted to determine the relative expression patterns of podocin, CX3CL1, nephrin, and WT1. **D** Representative images of podocyte ultrastructure in kidney tissues following cisplatin injection, as shown in TEM. Scale bar = 500 nm. **E** Representative micrographs of CX3CL1 and nephrin staining in kidney tissues. Scale bar = 10 μm. (WT, wild type; KO, knockdown; H&E, hematoxylin and eosin; PAS, periodic acid-Schiff; BUN, blood urea nitrogen; Scr, serum creatinine; WT1, Wilms tumor protein; TEM, transmission electron microscopy; *p* value was calculated by one-way analysis of variance and Tukey’s test. *p < 0.05, **p < 0.01, and ***p < 0.001 vs. the control group; ^#^*p* < 0.05, ^##^*p* < 0.01, and ^###^*p* < 0.001 vs. the Cis group)

We first quantified the expression levels of the chemokine CX3CL1 in the kidneys of Cis-AKI mice. An increase in CX3CL1 levels was observed in the kidneys of mice treated with cisplatin, as illustrated in Fig. 1E. Histological analysis of glomeruli indicated that the cisplatin group exhibited a higher number of inflammatory cells and increased glomerular sclerosis scores compared to the control group, partially restored by CX3CL1 knockout (Fig. 1A). Moreover, Scr and BUN levels were significantly elevated in the cisplatin group compared to the control group, and this effect was mitigated by CX3CL1 knockdown (Fig. 1B). We further assessed podocyte injury in vivo, revealing that the cisplatin group displayed elevated expression of podocyte-specific proteins, namely podocin, nephrin, and WT1, in contrast to the control (Fig. 1C). Morphological analysis revealed podocyte injury in the cisplatin group, characterized by glomerular basement membrane (GBM) thickening and podocyte foot process broadening and effacement (Fig. 1D). Additionally, the localization of CX3CL1 was enhanced while that of nephrin was reduced in the cisplatin group compared to the control (Fig. 1E). Notably, these alterations were reversed in CX3CL1-KO mice. Taken together, these results suggest that CX3CL1 deficiency could alleviate kidney injury and mitigate podocyte dysfunction in Cis-AKI mice.

### CX3CL1 deficiency alleviated cisplatin-induced mouse kidney ferroptosis

**Fig. 2:**
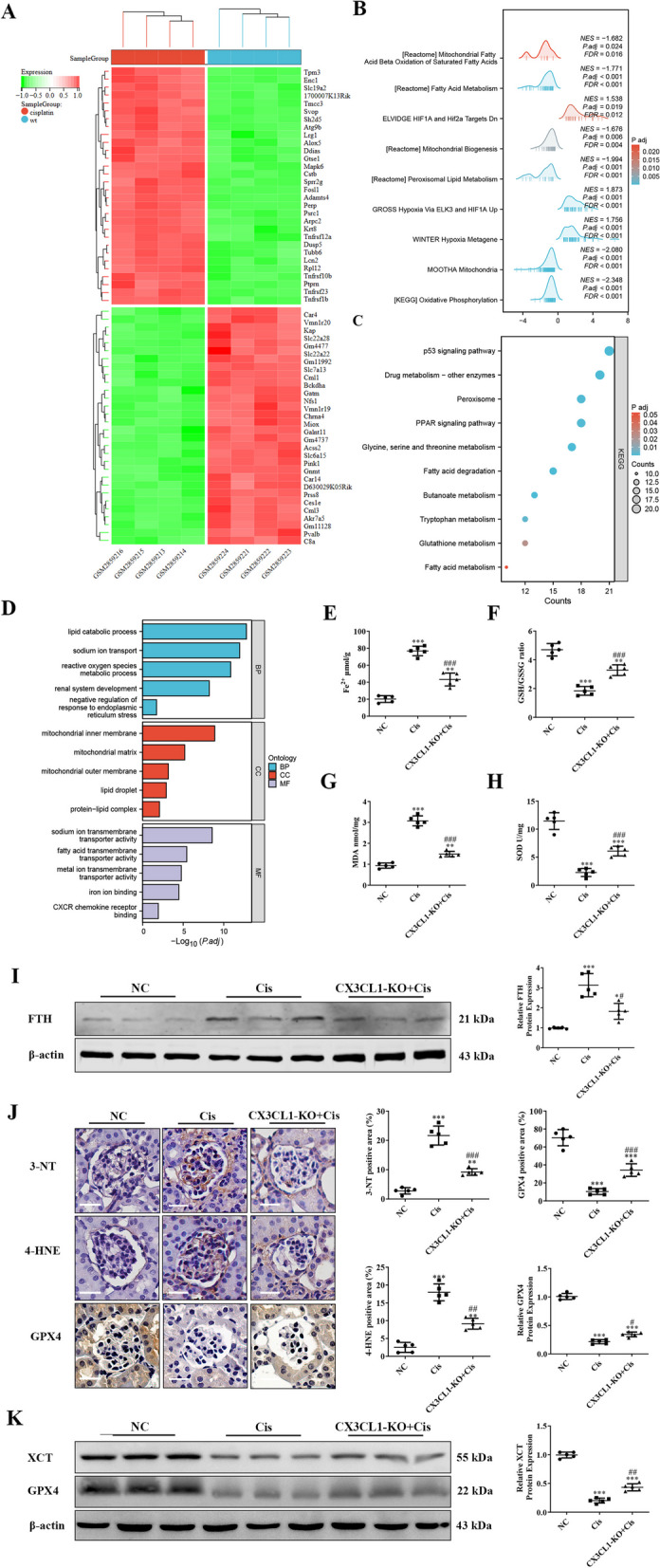
CX3CL1 deficiency reduced ferroptosis in renal tissues with cisplatin-induced AKI. **A**–**D** RNA sequencing analysis. **E**–**H** Measurement of Fe^2+^, GSH/GSSG ratio, MDA, and SOD levels in kidney tissues. **I** Expression of ferritin heavy chain in kidney tissues, as measured using western blotting. **J** Representative images displaying the expression of 3-NT, 4-HNE, and GPX4 as determined by immunohistochemistry. Scale bar = 20 μm. **K** Western blotting analysis of GPX4 and XCT expression profiles. (AKI, acute kidney injury; GSH/GSSG, glutathione/oxidized glutathione; MDA, malondialdehyde; SOD, superoxide dismutase; 3-NT, 3-nitrotyrosine; 4-HNE, 4-Hydroxynonenal; GPX4, glutathione peroxidase 4; XCT, solute carrier family 7 member 11; *P* value was calculated by one-way analysis of variance and Tukey’s test. *p < 0.05, **p < 0.01, and ***p < 0.001 vs. the control group; ^#^*p* < 0.05, ^##^*p* < 0.01, and ^###^*p* < 0.001 compared to the Cis group)

Transcriptome sequencing data were utilized to examine changes in mouse kidney tissue following cisplatin treatment. In cisplatin-treated WT mice, 617 genes were substantially downregulated, while 644 genes were upregulated compared to controls. The top 20 genes with the most significant upregulation were visualized in a heat map (Fig. 2A), encompassing genes related to metabolism, endoplasmic reticulum activity, oxidative stress, and ferroptosis. GO and KEGG analyses of differentially expressed genes indicated significant enrichment in metabolism-related pathways (Fig. 2B, C). GSEA analysis further revealed substantial alterations in fatty acid metabolism, Mitochondrial Biogenesis, and Hypoxia processes in cisplatin-treated kidneys (Fig. 2D). RNA-seq analysis substantiated the link between ferroptosis-related oxidative stress and lipid metabolism in Cis-AKI.

Given the significant role of iron overload in ferroptosis, we quantified serum and kidney iron content. Cisplatin treatment led to elevated iron levels in both serum and renal tissues, which was mitigated by CX3CL1 deficiency treatment (Fig. 2E, I). Another hallmark of ferroptosis is the depletion of GSH and accumulation of lipid peroxide. In contrast to controls, cisplatin-treated mice displayed an increased GSH/GSSG ratio in kidneys (Fig. 2F), which was reversed by CX3CL1 knockout. Regarding lipid peroxidation biomarkers, higher levels of MDA, 3-NT, and 4-HNE, along with decreased SOD levels, were detected in cisplatin-treated kidneys compared to controls. These effects were attenuated by CX3CL1 knockout (Fig. 2G, H, J). We further explored the impact of CX3CL1 knockout on kidney ferroptosis in cisplatin-treated mice by examining the expression profiles of the ferroptosis biomarkers XCT (cystine/glutamate antiporter) and GPX4 (a well-known ferroptosis regulator). As expected, CX3CL1 knockout reversed cisplatin-induced upregulation of mouse kidney XCT and GPX4 expression levels (Fig. 2K).

### CX3CL1 inhibition prevented cisplatin-induced podocyte ferroptosis

**Fig. 3:**
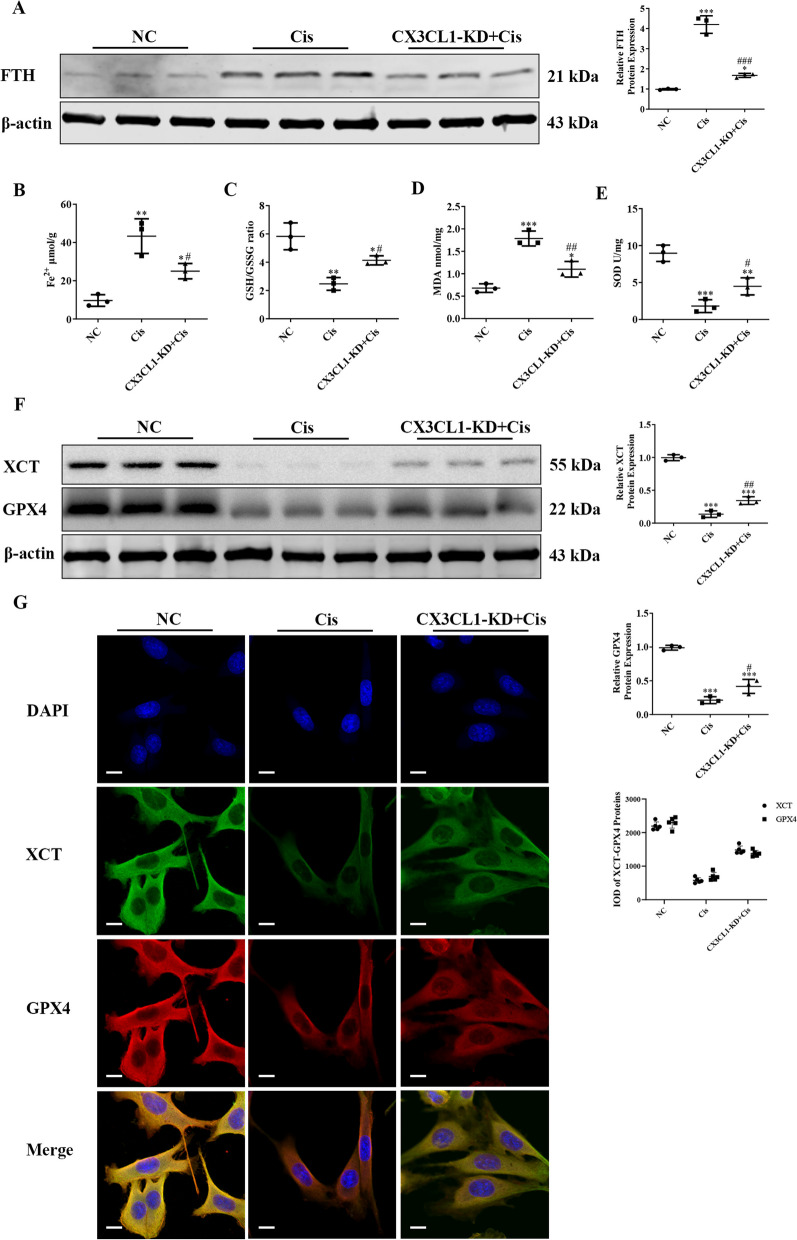
CX3CL1 knockdown inhibited cisplatin-induced ferroptosis in podocytes. **A** Expression of ferritin heavy chain in podocytes measured through western blotting. **B**–**E** The levels of Fe^2+^, GSH/GSSG ratio, MDA, and SOD were measured in podocytes. **F** Evaluation of XCT and GPX4 expression profiles in podocytes using western blotting. **G** Representative micrographs of podocytes stained with GPX4 and XCT. Scale bar = 10 μm. (GSH/GSSG, glutathione/oxidized glutathione; MDA, malondialdehyde; SOD, superoxide dismutase; XCT, solute carrier family 7 member 11; GPX4, glutathione peroxidase 4; *p* value was calculated by one-way analysis of variance and Tukey’s test. *p < 0.05, **p < 0.01, and ***p < 0.001 vs. the control group; ^#^*p* < 0.05, ^##^*p* < 0.01, and ^###^*p* < 0.001 vs. the Cis group)

We next investigated whether CX3CL1 knockdown could protect podocytes against cisplatin-induced ferroptosis. Accordingly, we measured iron content in podocytes. Western blot analysis indicated elevated levels of the heavy ferritin chain (FTH) in cisplatin-treated podocytes compared to controls; CX3CL1 knockdown mitigated this effect (Fig. 3A). Additionally, CX3CL1 knockdown mitigated the cisplatin-induced upregulation of Fe^2+^ and MDA levels and downregulation of SOD level and GSH/GSSG ratio in podocytes (Fig. 3B–E). Furthermore, western blotting and immunofluorescence assays revealed that GPX4 and XCT levels and their localization were reduced in cisplatin-treated podocytes and then elevated upon CX3CL1 knockdown (Fig. 3F, G).

### CX3CL1 deficiency improved cisplatin-induced mouse kidney and podocyte mitochondrial dysfunction

**Fig. 4:**
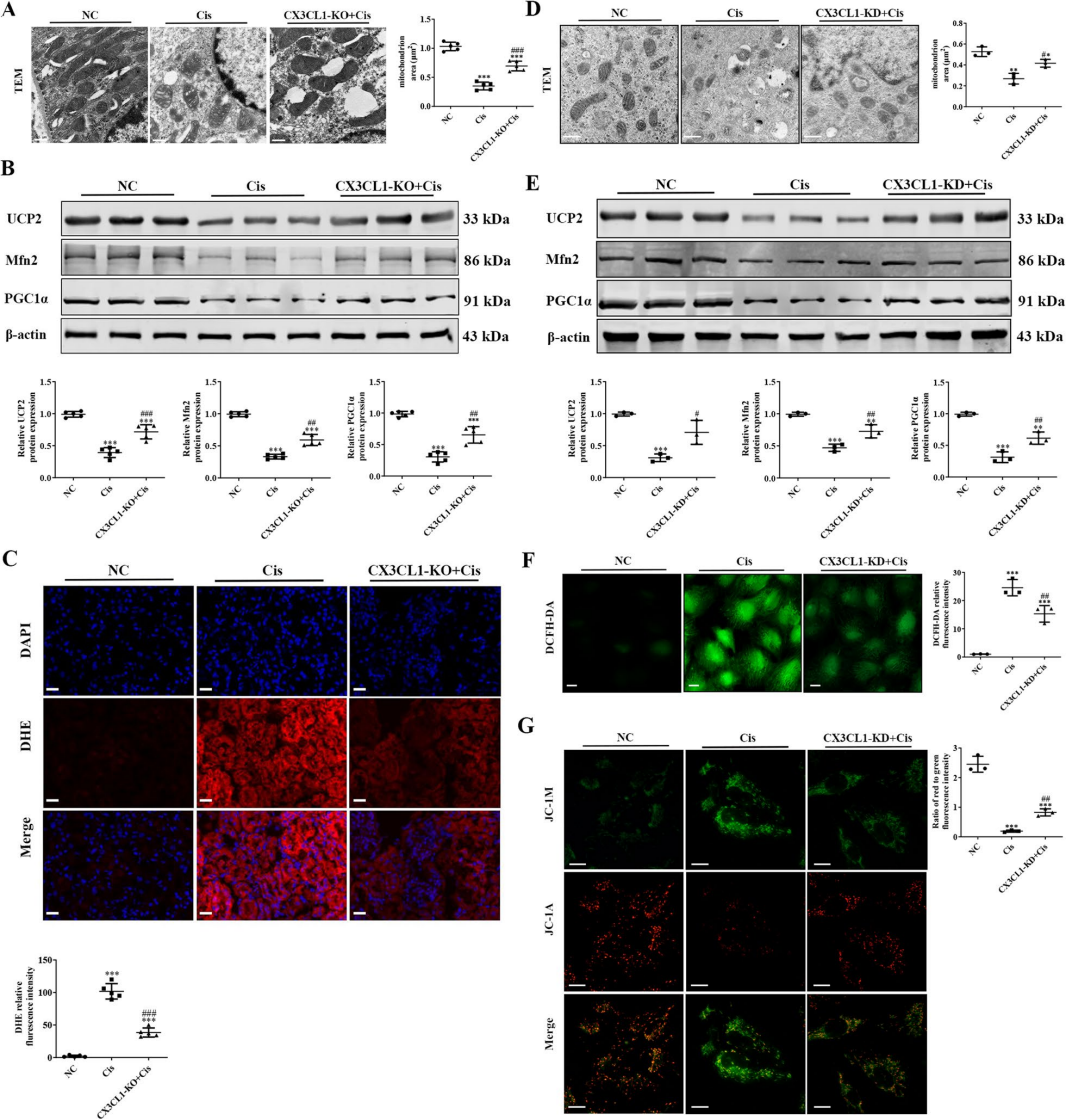
The mitochondrial function was improved by CX3CL1 deficiency in mice with cisplatin-induced AKI. **A** TEM images of renal tissues were captured. Scale bar = 500 nm. **B** The relative expression profiles of the mitochondrial proteins UCP2, Mfn2, and PGC1α in kidney tissues were determined by Western blotting. **C** DHE staining was conducted to determine the ROS level in renal tissues. Scale bar = 20 μm. **D** TEM was employed to capture images of podocytes. Scale bar = 500 nm. **E** Western blot analysis illustrating the relative expression patterns of the mitochondrial proteins UCP2, Mfn2, and PGC1α in podocytes. **F** The ROS level was detected through DCFH-DA labeling. Scale bar = 10 μm. **G** JC-1 staining was conducted to detect MMP in podocytes. Scale bar = 10 μm. (AKI, acute kidney injury; TEM, transmission electron microscopy; UCP2, uncoupling protein 2; Mfn2, Mitofusin 2; PGC1α, peroxisome proliferators-activated receptor γ coactivator l-alpha; DHE, dihydroethidium; ROS, reactive oxygen species; DCFH-DA, 2,7-dichlorodihydrofluorescein diacetate; MMP, matrix metalloproteinase; *P* value was calculated by one-way analysis of variance and Tukey’s test. *p < 0.05, **p < 0.01, and ***p < 0.001 vs the control group; ^#^*p* < 0.05, ^##^*p* < 0.01, and ^###^*p* < 0.001 vs the Cis group)

It is well-established that mitochondria play a pivotal role in maintaining cellular homeostasis. Mitochondria undergo various morphological changes during ferroptosis, including size reduction, heightened membrane density, and diminished or absent mitochondrial ridges. In the present study, TEM images revealed shrunken mitochondria, increased mitochondrial membrane density, and absence of mitochondrial cristae in the cisplatin group compared to the control group (Fig. 4A). These changes were reversed upon CX3CL1 knockout. Mitochondria reportedly serve as hubs for cell metabolism, redox reactions, and signaling. The mitochondrial biogenesis-associated proteins UCP and Mfn2 play crucial roles (Zhou et al. 2022). Western blot analysis demonstrated that CX3CL1 knockout led to elevated UCP2, Mfn2, and PGC1α levels in kidney tissues from the cisplatin group (Fig. 4B). Furthermore, we examined ROS production in mouse kidneys, given that ROS accumulation is central to ferroptosis. Indeed, it is widely acknowledged that excessive ROS production can impact mitochondrial morphology (Dasari and Tchounwou 2014). Utilizing a DHE probe, we monitored ROS production in kidney tissues. Cisplatin treatment triggered rapid ROS production in mouse kidney tissues, which could be mitigated by CX3CL1 knockout (Fig. 4C). Additionally, we explored whether CX3CL1 knockdown could protect podocytes against cisplatin-induced mitochondrial injury and ROS production. Electron microscopy revealed mitochondrial injury in cisplatin-treated podocytes, evidenced by swelling, reduced volume, and decreased cristae. These effects were reversed upon CX3CL1 knockdown (Fig. 4D). Similarly, we quantified mitochondrial biogenesis proteins in podocytes. Western blot analysis indicated that CX3CL1 knockdown increased UCP2, Mfn2, and PGC1α levels in cisplatin-treated podocytes (Fig. 4E). Moreover, cisplatin treatment markedly elevated ROS production in podocytes, which was countered by CX3CL1 knockdown (Fig. 4F). The mitochondrial membrane potential (MMP) assay revealed that normal podocytes displayed red fluorescence due to JC-1 polymerization within mitochondria. However, exposure of these podocytes to cisplatin led to an upregulation in ΔΨm dissipation, resulting in green fluorescence due to monomeric JC-1 in mitochondria (Fig. 4G). These findings suggest that CX3CL1 deficiency counters cisplatin-induced mitochondrial dysfunction in mouse kidneys and podocytes.

### CX3CL1 deficiency attenuated cisplatin-induced mouse kidney and podocyte inflammation

**Fig. 5:**
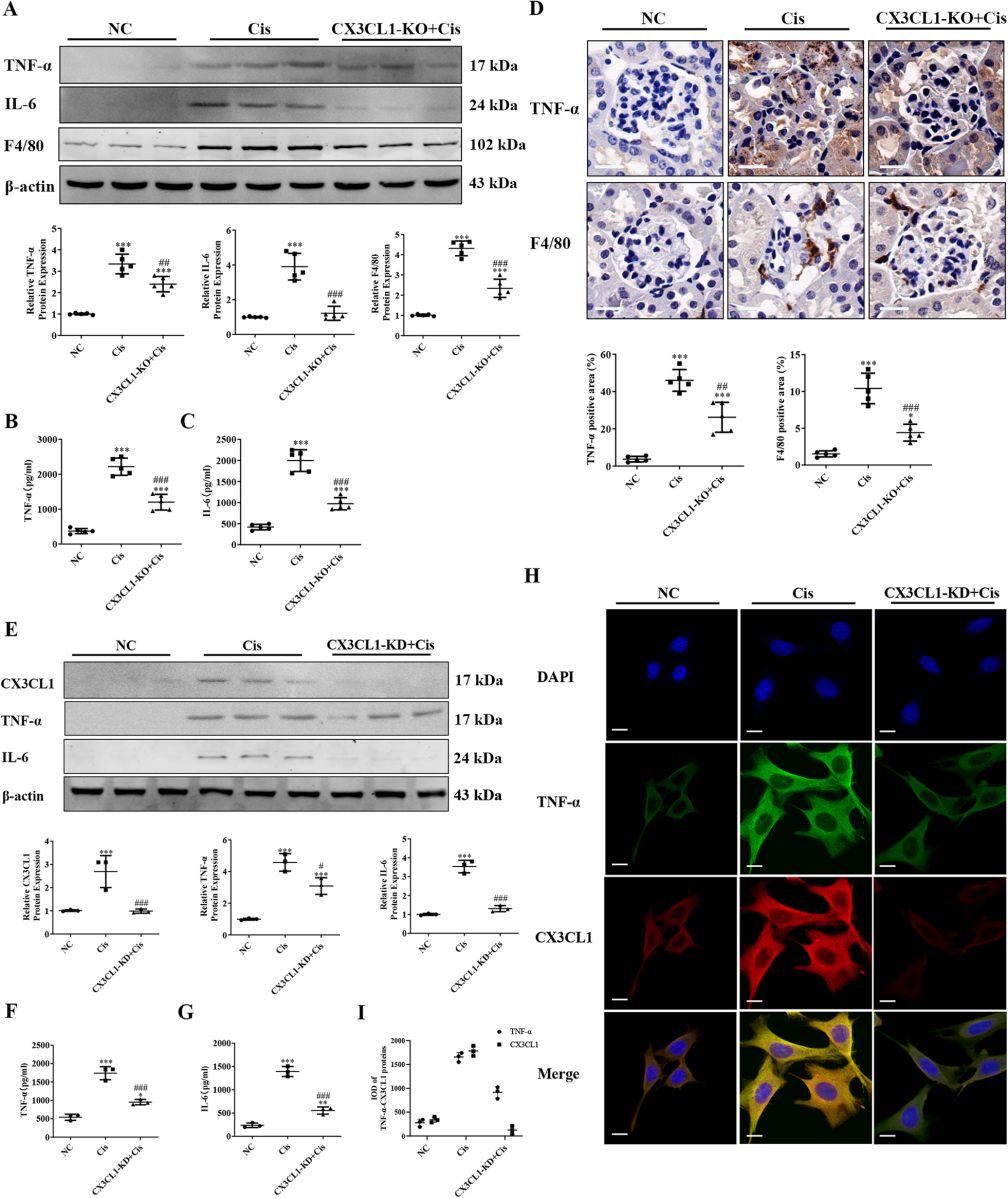
CX3CL1 deficiency attenuated kidney inflammation and macrophage infiltration in cisplatin-induced model mice. **A** Western blotting was conducted to assess the expression profiles of IL-6, TNF-α, and F4/80 in kidney tissues. **B**, **C** Levels of IL-6 and TNF-α in mouse sera. **D** Image representations of TNF-α and F4/80 expression by immunohistochemistry. Scale bar = 20 μm. **E** podocytes were transfected with siRNA-CX3CL1 as indicated and treated with cisplatin (10 μM) for 24 h. CX3CL1, TNF-α, and IL-6 expression profiles were then measured by western blotting. **F**, **G** Levels of IL-6 and TNF-α in podocyte cell supernatant. **H**, **I** Representative micrographs of CX3CL1 and TNF-α staining in podocytes. Scale bar = 10 μm. (IL-6, interleukin 6; TNF-α, tumor necrosis factor-α; *P* value was calculated by one-way analysis of variance and Tukey’s test. *p < 0.05, **p < 0.01, and ***p < 0.001 vs the control group; ^#^*p* < 0.05, ^##^*p* < 0.01, and ^###^*p* < 0.001 vs the Cis group)

We examined whether CX3CL1 inhibition could curb the inflammatory responses induced by cisplatin in mouse kidneys and podocytes. In contrast to controls, the cisplatin group exhibited elevated serum levels of TNF-α and IL-6 (Fig. 5A–C), along with enhanced renal localization of TNF-α (Fig. 5D). However, the elevation of IL-6 and TNF-α caused by cisplatin was mitigated by CX3CL1 deficiency. Additionally, we evaluated how CX3CL1 knockdown influenced macrophage infiltration into the kidneys. The macrophage biomarker F4/80 was observed in the kidneys of mice treated with cisplatin and reduced by CX3CL1 knockdown (Fig. 5A, D). In vitro investigations were conducted to assess the impact of CX3CL1 knockdown on the inflammatory process induced by cisplatin in podocytes. Treatment with cisplatin led to higher levels of TNF-α, IL-6, and CX3CL1 expression in podocytes, an elevation that was reversed by CX3CL1 knockdown (Fig. 5E–G). Furthermore, an immunofluorescence assay unveiled notable CX3CL1 and TNF-α localization in the cytoplasm of podocytes, an effect reversed by CX3CL1 knockdown (Fig. 5H, I). These findings underscore that CX3CL1 inhibition protects against cisplatin-induced kidney inflammation by curbing podocyte inflammatory response.

### CX3CL1 deficiency alleviated cisplatin-induced ER stress and HIF1A/HO-1 signal activation in mouse kidney and podocytes

**Fig. 6:**
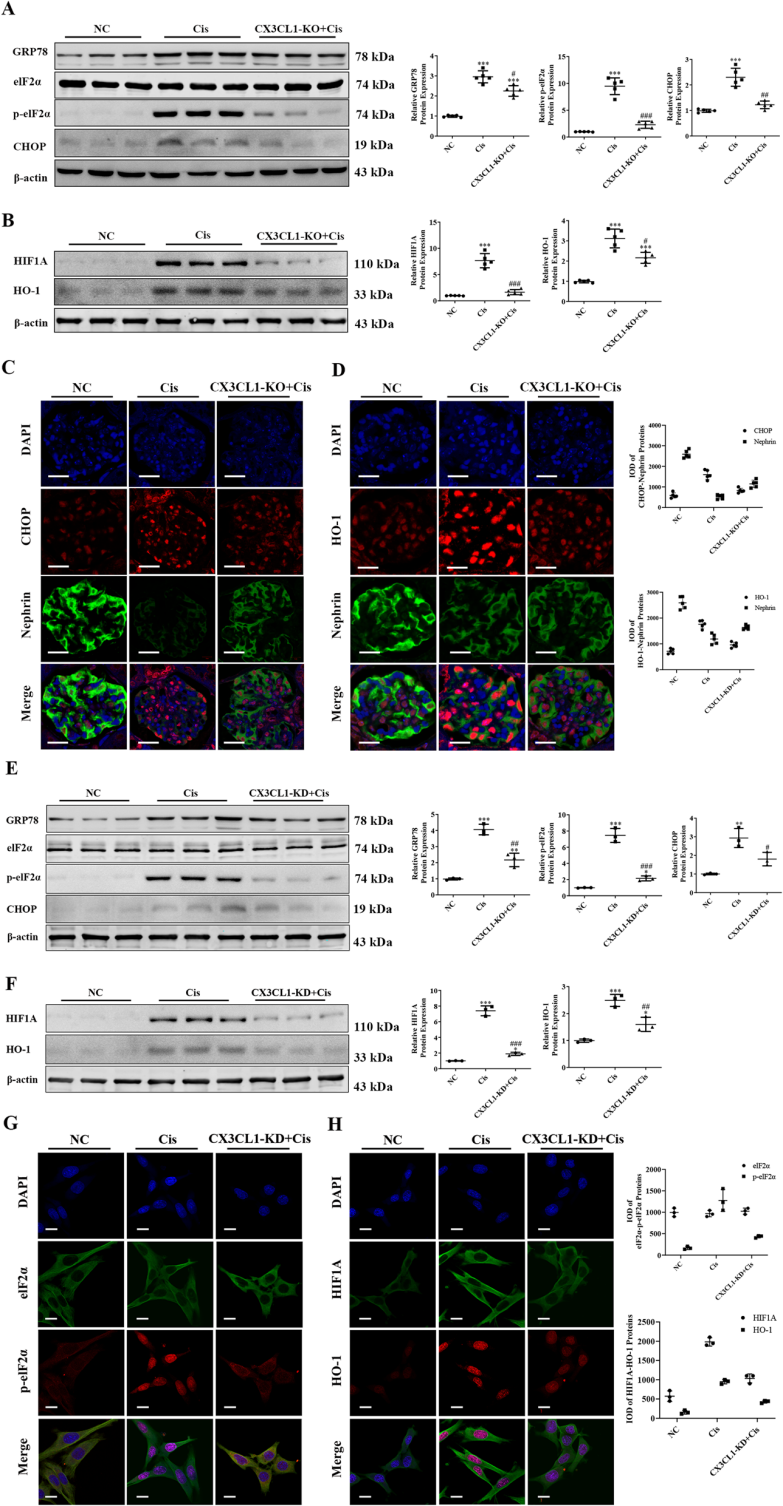
CX3CL1 deficiency inhibited cisplatin-induced ER stress and HIF1A/HO-1 signal activation in kidney tissues. **A** Western blot analysis of proteins in kidney tissues that are associated with ER stress. **B** Western blotting was conducted to evaluate the HIF1A and HO-1 protein expression patterns in renal tissues. **C** Representative micrographs of CHOP and nephrin staining in renal tissues. Scale bar = 10 μm. **D** Representative micrographs of HO-1 and nephrin staining in renal tissues. Scale bar = 10 μm. **E** Western blot analysis was conducted to analyze the podocyte expression profiles of proteins associated with ER stress. **F** Podocytes’ HIF1A and HO-1 expression profiles were evaluated by western blotting. **G** Representative micrographs of podocytes stained with eIF2α and p-eIF2α. Scale bar = 10 μm. **H** Representative micrographs of podocytes stained with HIF1A and HO-1. Scale bar = 10 μm. (ER, endoplasmic reticulum; HIF1A/HO-1, hypoxia inducible factor 1-alpha/heme oxygenase-1; CHOP, CCAAT enhancer binding protein-homologous protein; eIF2α, eukaryotic translation initiation factor 2A; *P* value was calculated by one-way analysis of variance and Tukey’s test. *p < 0.05, **p < 0.01, and ***p < 0.001 vs the control group; ^#^*p* < 0.05, ^##^*p* < 0.01, and ^###^*p* < 0.001 vs the Cis group)

It is now understood that ER stress proteins significantly influence nephrotoxicity (Deng et al. 2021). Herein, we observed that cisplatin treatment induced the expression of ER stress-associated proteins GRP78, p-eIF2α, and CHOP, an effect that was reversed by CX3CL1 knockout (Fig. 6A, C). Additionally, we measured HIF1A and HO-1 levels in kidneys based on reports that HO-1 plays a crucial role in ferroptosis and cellular stress (Holditch et al. 2019), and HIF1A can induce HO-1 expression to regulate oxidative stress (Peti-Peterdi and Sipos 2010). CX3CL1 knockout countered the upregulation of HIF1A and HO-1 induced by cisplatin (Fig. 6B, D). In vitro experiments were performed to evaluate the impact of CX3CL1 knockdown on ER stress-associated proteins and HIF1A/HO-1 signaling induced by cisplatin in podocytes. Increased levels of ER stress-associated proteins p-eIF2α, GRP78, and CHOP, as well as enhanced p-eIF2α protein localization, were observed in cisplatin-treated podocytes; these effects were mitigated by CX3CL1 knockdown (Fig. 6E, G). Furthermore, we assessed whether CX3CL1 knockdown curbs HIF1A/HO-1 signaling induced by cisplatin treatment in podocytes. Western blot analysis and immunofluorescence assay demonstrated that cisplatin treatment led to upregulated HIF1A and HO-1 expression in podocytes, with CX3CL1 knockdown reversing these effects (Fig. 6F, H). These results suggest that CX3CL1 deficiency could alleviate ER stress activation and the HIF1A/HO-1 signaling pathway, thereby mitigating ferroptosis in cisplatin-induced kidney dysfunction and podocytes.

### CX3CL1 inhibition reduced podocyte injury caused by cisplatin exposure by alleviating ER stress

**Fig. 7:**
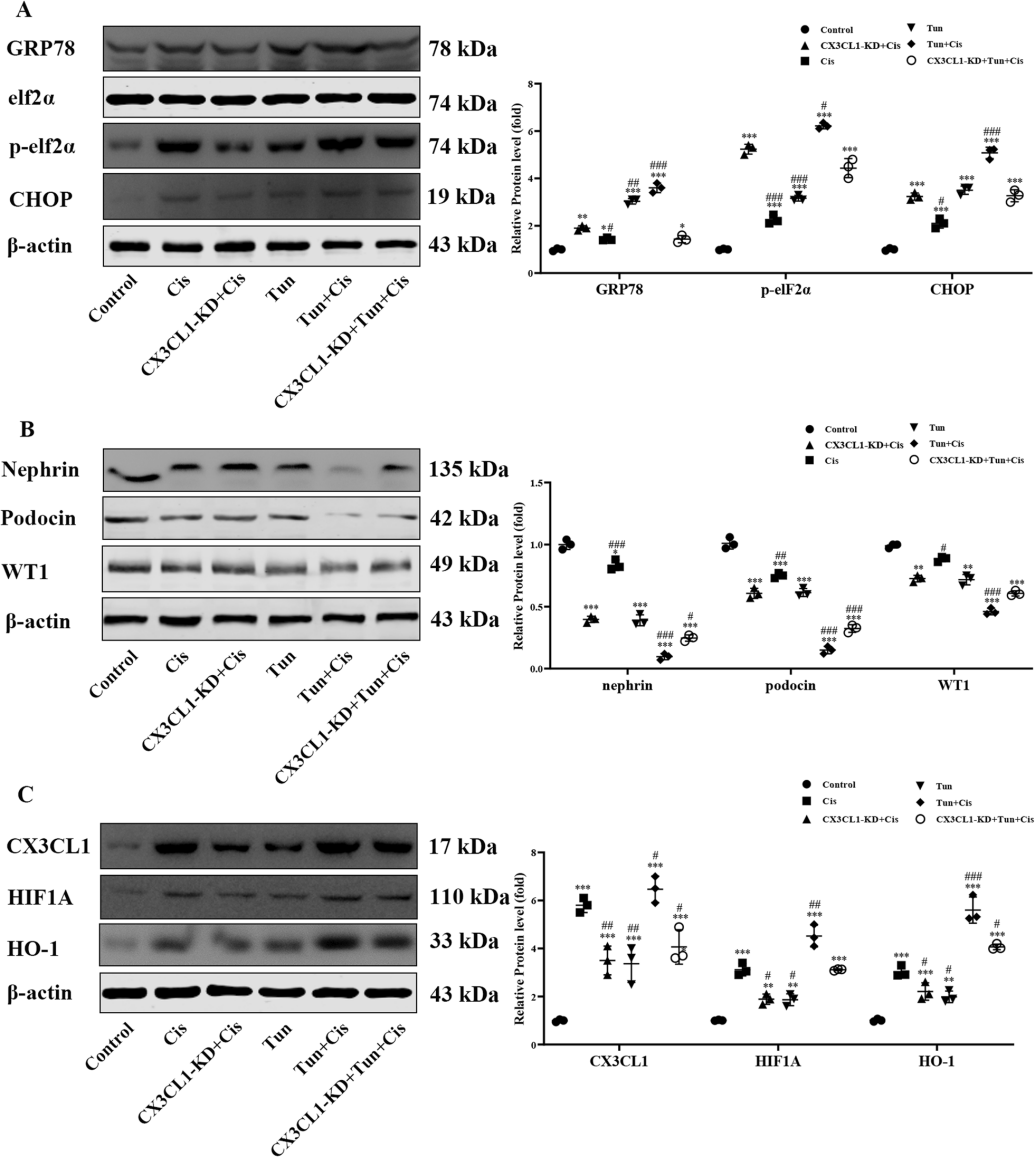
CX3CL1 reduced ER stress, which in turn alleviated cisplatin-induced podocyte damage. **A** The protein expression levels associated with ER stress were evaluated by Western blotting. **B** The levels of podocyte-specific proteins were evaluated by Western blotting. **C** CX3CL1, HIF1A, and HO-1 levels were evaluated by Western blot. (ER, endoplasmic reticulum; HIF1A, hypoxia inducible factor 1-alpha; HO-1, heme oxygenase-1; *P* value was calculated by one-way analysis of variance and Tukey’s test. *p < 0.05, **p < 0.01, and ***p < 0.001 vs the control group; ^#^*p* < 0.05, ^##^*p* < 0.01, and ^###^*p* < 0.001 vs the Cis group)

We next investigated the relationship between the protective effect of CX3CL1 knockdown and the induction of ER stress in cisplatin-treated podocytes. Western blot analysis revealed that the ER stress pathway activator tunicamycin (Tun, 5 μg·mL^−1^) reactivated ER stress initially suppressed by CX3CL1 knockdown in cisplatin-treated podocytes (Fig. 7A), reversing the effects of CX3CL1 knockdown on podocyte injury protection and HIF1A/HO-1 activation (Fig. 7B, C). These findings suggest that CX3CL1 suppression confers protective effects to some extent against cisplatin-induced podocyte damage via mitigating ER stress pathway activity.

## Discussion

Herein, we substantiated that targeted inhibition of the chemokine CX3CL1 could effectively modulate podocyte ferroptosis, thereby conferring protection against Cis-AKI in mice. Our mechanistic insights suggest that the inhibition of CX3CL1 might attenuate ferroptosis by targeting the ER stress pathway and the HIF1A/HO-1 signaling pathway.

Cisplatin is a widely used first-line chemotherapeutic agent for various malignancies, such as lymphoma, lung, bladder, and ovarian cancers (Dasari and Tchounwou 2014). Current evidence suggests that Cisplatin easily penetrates the glomerular filtration barrier (Deng et al. 2021), leading to localized cisplatin accumulation and nephrotoxicity. Accordingly, elevated Scr and BUN levels, along with inflammatory cell infiltration and renal tissue fibrosis, can indicate severe renal injury resulting from cisplatin accumulation (Holditch et al. 2019). Podocytes, integral components of the glomerular basement membrane, play a crucial role in glomerular filtration balance through podocyte foot process motility (Peti-Peterdi and Sipos 2010). Indeed, these terminally differentiated cells cannot regenerate, rendering them susceptible to injury (Assady et al. 2017; Schell and Huber 2017). Disturbed podocyte process maintenance, abnormal filopodia formation, and impaired glomerular filtration barrier maintenance are typically observed in conditions like IgA nephropathy, diabetic nephropathy, and acute kidney injury (Liu et al. 2017; Lee et al. 2020). In this context, our study observed elevated renal markers, such as Scr and BUN, along with glomerulosclerosis and inflammatory infiltration in mice treated with cisplatin. Correspondingly, compared to controls, elevated TNF-α, IL-6, and F4/80 expression levels were noted in cisplatin-treated podocytes. These findings validated the successful establishment of Cis-AKI mouse and podocyte injury models.

Inflammatory chemokines play a pivotal role in orchestrating leukocyte attraction, migration, and differentiation within injured kidneys. The present study centered on CX3CL1 due to its marked upregulation in renal diseases. CX3CL1 is expressed in various renal cell types, including podocytes, endothelium, tubular epithelium, mesangial, and renal cancer cells (Zhuang et al. 2017; Kim et al. 2011). Nakatani et al. documented elevated CX3CL1 expression in systemic lupus erythematosus, correlating with lupus severity in animal models (Nakatani et al. 2010). Song et al. demonstrated that CX3CL1 deficiency reduced extracellular matrix deposition in a diabetic nephropathy mouse model (Song et al. 2013). Interestingly, in our current study, CX3CL1 levels were elevated in both the Cis-AKI model and the cisplatin-induced podocyte injury model. Notably, recent investigations have highlighted the protective potential of CX3CL1 inhibition against kidney inflammation. Oh et al. showed that CX3CL1 inhibition had a protective effect against ischemic acute kidney failure (Oh et al. 2008), while Yong et al. found that CX3CL1 deficiency protects against fructose-induced kidney injury (Yu et al. 2018). Our earlier work consistently demonstrated the impact of CX3CL1 deficiency on lipopolysaccharide-induced AKI (Gong et al. 2021, 2022). Herein, we observed that CX3CL1 deficiency reduced cisplatin-induced dysfunction, inflammatory factor aggregation, and glomerulosclerosis in the kidneys of Cis-AKI model mice. Moreover, CX3CL1 deficiency led to decreased inflammatory cytokine levels in cisplatin-induced podocytes compared to control podocytes. These findings firmly underscore the therapeutic potential of CX3CL1 deficiency against Cis-AKI and podocyte injury.

Although previous studies have extensively explored the association between Cis-AKI and cell death mechanisms like apoptosis and necrosis (Parisis et al. 2019; Al-Salam et al. 2021), the relationship between ferroptosis and Cis-AKI has been relatively underexplored. It is acknowledged that cisplatin triggers the accumulation of free iron (Baliga et al. 1998). It is now understood that the hallmark of ferroptosis is the accumulation of lipid ROS due to iron-dependent lipid peroxidation (Mou et al. 2019). The significance of the XCT-GSH-GPx4 axis in the ferroptosis process is noteworthy. It has been reported that GSH depletion can also lead to lipid peroxidation, and impaired cysteine uptake, facilitated by the amino acid antiporter XCT, can reduce GPX4 activity (Yang and Stockwell 2016). GPX4, an enzyme critical for lipid ROS regulation, converts GSH into glutathione disulfide (GSSG) through an oxidation process, thus contributing to lipid peroxidation (Liu et al. 2021; Hadian and Stockwell 2020). Our investigation revealed elevated levels of total iron and lipid peroxidation in cisplatin-induced mouse kidneys and podocytes, validating the successful establishment of the cisplatin-induced podocyte injury and Cis-AKI mouse models. Drawing from our prior research findings and sequencing data analysis, we hypothesized that CX3CL1 deficiency plays a protective role in Cis-AKI model mouse kidneys and cisplatin-induced podocyte models by modulating ferroptosis. CX3CL1 knockout could ameliorate the depletion of XCT and GPX4 activities, alterations in lipid peroxidation, ROS levels, iron content, and the GSH/GSSG ratio in Cis-AKI model mouse kidneys and cisplatin-induced podocyte models. These results emphasize the regulatory influence of CX3CL1 deficiency on the XCT-GSH-GPx4 axis, providing the foothold for further research into anti-ferroptosis therapy for Cis-AKI.

Mitochondria have intricate roles encompassing iron utilization, metabolism, and energy production within cells. Eukaryotic cells convert cytosolic iron into heme and iron-sulfur (Fe/S) clusters after its uptake by mitochondria (Wang et al. 2020). Notably, Gao et al. demonstrated that mitochondrion-deficient cells were not susceptible to erastin-induced ferroptosis, highlighting the significance of mitochondria in this process (Gao et al. 2019). Additionally, Fang et al. revealed that inhibiting mitochondrial ROS production could effectively prevent glutathione-dependent ferroptosis (Fang et al. 2019). Beyond their role in ferroptosis regulation, mitochondria also govern inflammatory cytokines. Zhao et al. underscored the link between decreased mitochondrial ROS levels and the inhibition of TNF-α and IL-6 production in AKI (Zhao et al. 2021). Our study observed decreased or absent mitochondrial cristae, heightened mitochondrial membrane density, and reduced mitochondrial volume in cisplatin-induced model mouse renal tissues and podocytes. CX3CL1 deficiency mitigated mitochondrial injury in mouse renal tissues and podocytes following cisplatin exposure. These insights strongly suggest that CX3CL1 deficiency could mitigate inflammation and ferroptosis in Cis-AKI model mouse kidneys and cisplatin-induced model podocytes through mitochondrial pathways.

It has been reported that HIF1A plays a pivotal role in eliciting responses to oxidative stress and is a primary target of endogenous cellular ROS. It influences cellular inflammation, metabolism, and stress responses (Otterbein et al. 2003). Notably, HIF1A has been implicated in regulating ferroptosis by inducing HO-1 expression (Chiang et al. 2018). HO-1, an ER microsome-anchored enzyme, plays a critical role in maintaining cellular homeostasis under oxidative stress (Gottlieb et al. 2012). Moreover, activated HO-1 leads to iron overload, culminating in ferroptosis due to ROS production (Chang et al. 2018). Instances of elevated HIF-1A and HO-1 levels in diabetic kidneys and their connection to ferroptosis have been reported (Feng et al. 2021). Wei et al. discovered that HO-1 activation mediates digitonin C-induced colorectal cancer (CRC) cell ferroptosis (Wei et al. 2021a). In the present study, we provided compelling evidence that cisplatin could induce elevated levels of HIF1A and HO-1 in mouse kidneys and podocytes, contributing to ferroptosis. However, CX3CL1 deficiency countered these effects by reducing HIF1A and HO-1 levels, alleviating cisplatin-induced ferroptosis phenotypes both in vivo and in vitro*.*

A clear association has been established between ER stress and ROS generation (Zeeshan et al. 2016). It is widely acknowledged that ER's primary role is to fold, modify, and mature proteins. When the equilibrium is disturbed, misfolded and unfolded proteins disrupt ER function, leading to ER stress (Hughes et al. 2017). Prolonged ER stress triggers GRP78 and eIF2α activation, subsequently increasing CHOP expression, culminating in cell injury. The protective effects of curcumin on renal function mediated by ER stress suppression have been validated by Yu et al. (2020). Furthermore, Wei et al. demonstrated that ginseng polysaccharides could prevent kidney cell ER stress by regulating PERK-eIF2α-ATF4 signaling (Wei et al. 2021b). In our study, ER stress-associated GRP78/eIF2α/CHOP proteins were upregulated in cisplatin-induced mouse kidneys and podocytes compared to control samples, signifying cisplatin-induced ER stress. Notably, CX3CL1 deficiency reduced the expression of ER stress-associated proteins. Intriguingly, the ER stress inducer tunicamycin reversed the effects of CX3CL1 inhibition, specifically enhancing the expression of ferroptosis-related proteins and HIF1A/HO-1 signaling, thereby inducing podocyte injury. These findings corroborate that CX3CL1 deficiency-induced protective effects against cisplatin-induced podocyte injury are attributed to inhibition of the ER stress pathway.

Certain limitations of the present study should be acknowledged. Indeed, acute kidney injury impacts a range of renal cells, including renal tubular epithelial cells, glomerular cells, renal macrophages, and interstitial cells. However, our in vitro experiments solely focused on podocytes. Future research should include other renal cell types to comprehensively assess the protective effects of CX3CL1 deficiency in Cis-AKI. Additionally, the potential of a CX3CL1 inhibitor as a novel treatment for cisplatin-induced kidney injury in clinical settings remains uncertain; its widespread implementation warrants thorough evaluation through large-scale clinical trials in the future.

## Conclusion

**Fig. 8:**
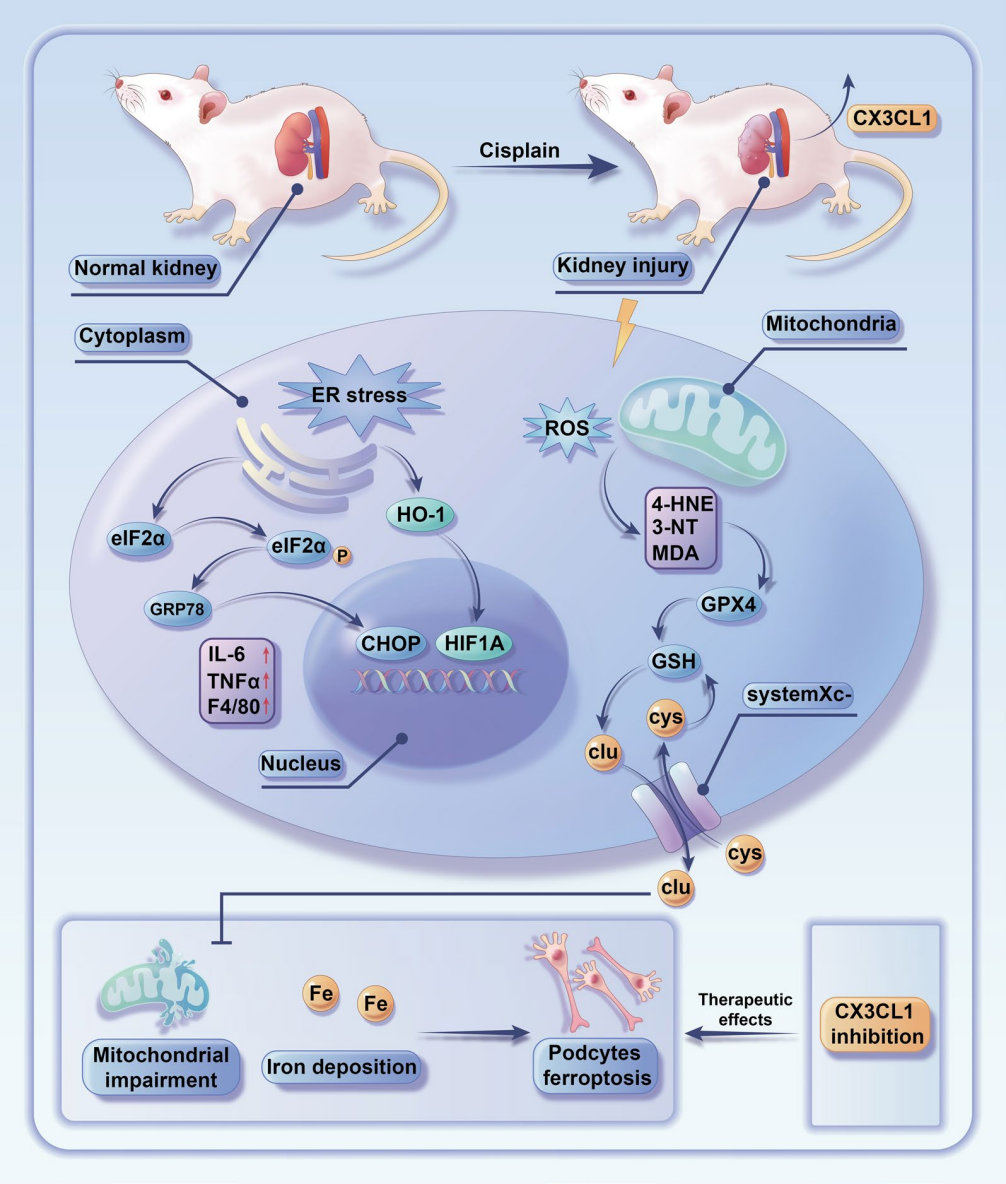
A proposed model for the CX3CL1 inhibition-mediated prevention of cisplatin-induced AKI by suppressing podocyte ferroptosis

In summary, our investigation delved into the underlying mechanisms of Cis-AKI. The implications of our findings are groundbreaking, indicating that CX3CL1 deficiency can mitigate podocyte ferroptosis through the modulation of ROS and ER stress (Fig. 8). This intervention alleviates Cis-AKI and stems from the inhibition of HIF1A/HO-1 signal activation. Our findings strongly advocate the viability of CX3CL1 deficiency as a promising approach for treating Cis-AKI.

## Data Availability

The data are available from the authors on reasonable request.

## Abbreviations

Cis-AKI: Cisplatin-induced acute kidney injury
HE: Hematoxylin–eosin
PAS: Periodate-Schiff
RNA-seq: RNA sequencing
Cre: Creatinine
BUN: Blood urea nitrogen
MDA: Malondialdehyde
ROS: Reactive oxygen species
SOD: Superoxide dismutase
GPX4: Glutathione peroxidase-4
ER: Endoplasmic reticulum
HO-1: Heme oxygenase-1
AKI: Acute kidney injury
CX3CL1-KO: CX3CL1-knockout
TPM: Total productive maintenance
GEO: Gene expression omnibus
GO: Gene ontology
KEGG: Kyoto encyclopedia of genes and genomes
GSEA: Gene set enrichment analysis
Scr: Serum creatinine
GSH/GSSG: Glutathione/oxidized glutathione
IL-6: Interleukin-6
TNF-α: Tumor necrosis factor-α
IHC: Immunohistochemistry
TEM: Transmission electron microscopy
IF: Immunofluorescence
PFA: Paraformaldehyde
MMP: Mitochondrial membrane potential
SD: Standard deviation
ANOVA: Analysis of variance
